# Mechanical Properties and Parameter Optimization for the “Suitable Harvest” Stage of Vegetable Sweet Potato Shoot Tips in Mechanized Harvesting

**DOI:** 10.3390/plants15071021

**Published:** 2026-03-26

**Authors:** Haiyang Shen, Oumeng Qiao, Gongpu Wang, Guangyu Xue, Wenqin Ding, Lianglong Hu, Guomin Zhou

**Affiliations:** 1 Agricultural Information Institute of CAAS, Beijing 100081, China; shenhaiyang@caas.cn; 2 Nanjing Institute of Agricultural Mechanization, Ministry of Agriculture and Rural Affairs, Nanjing 210014, China; qiaooumeng@caas.cn (O.Q.); wanggongpu@caas.cn (G.W.); 82101232111@caas.cn (G.X.); dingwenqin@caas.cn (W.D.); 3 Key Laboratory of Modern Agricultural Equipment, Ministry of Agriculture and Rural Affairs, Nanjing 210014, China; 4 College of Engineering, Nanjing Agricultural University, Nanjing 210031, China

**Keywords:** vegetable sweet potato shoot tip, suitable harvest stage, mechanical properties, shear force, mechanized harvesting parameters

## Abstract

Vegetable sweet potato shoot tips are harvested repeatedly for fresh markets, but harvest timing and cut length are still determined largely by experience, limiting their translation into mechanized design parameters and control thresholds. We conducted a two-factor shear-mechanics experiment using three cultivars (‘Fu 23’, ‘Fu 18’, and ‘HD-V4’) and five shoot-tip length levels (10–30 cm), while also measuring stem diameter and moisture content. Because shear tests were performed on short stem segments sampled from a fixed internodal position relative to the apex, the length factor is interpreted mainly as a field-operable harvest criterion and only secondarily as a variable partly associated with tissue position. Moisture content was uniformly high and did not differ among cultivars (*p* > 0.05). In a pooled two-way ANOVA, length significantly affected maximum shear force (*p* < 0.01), cultivar was also significant (*p* < 0.05), and the interaction was not significant (*p* > 0.05). After including stem diameter as a covariate, both diameter and length remained significant, whereas cultivar became non-significant, indicating that stem diameter explains much of the apparent cultivar difference in absolute load. The reported stress is nominal shear stress. Laboratory-based 95th percentile design loads with *γ* = 1.3 provide conservative engineering thresholds for preliminary design and harvest-window back-calculation.

## 1. Introduction

Sweet potato (*Ipomoea batatas* L.) is an important global food and economic crop, characterized by stable productivity, broad adaptability, and strong potential for diversified utilization, and it has long held strategic importance in food security, feed use, and processing industries [1,2]. According to FAO statistics data, in 2024 China’s sweet potato cultivation area was approximately 2.321 × 10^6^ ha, accounting for 32.17% of the global total, while total production reached 5.157 × 10^7^ t, representing 56.71% of global output; both planting area and total production have ranked first in the world for many years [3]. Beyond the traditional industry structure centered on storage-root utilization, vegetable sweet potato uses young stems and leaves, particularly shoot tips, as the main marketable product, thereby extending sweet potato utilization from a “storage-root-only” model to a coordinated “storage root + shoot” model and creating application scenarios such as fresh consumption, fresh-cut vegetable processing, and multiple harvests per season [4,5,6]. This utilization pattern not only helps improve annual output efficiency per unit area and industrial value addition, but also places higher demands on the harvesting stage, where attention must extend beyond yield to include harvest uniformity, operational efficiency, tissue damage control, and the stability of supply under repeated harvesting. In current production practice, decisions regarding when to harvest, what shoot-tip length to harvest, and how to balance efficiency and low damage still rely largely on empirical judgment, making it difficult to establish standardized parameters that are verifiable and transferable, and even more difficult to translate them directly into structural design criteria and control thresholds for mechanized harvesting equipment.

From the perspective of operating mechanisms, harvesting vegetable sweet potato shoot tips differs substantially from storage-root harvesting in both key processes and equipment requirements. Storage-root harvesting emphasizes soil excavation, root–soil separation, and low-damage conveying, whereas shoot-tip harvesting is centered on the stable control of cutting behavior in tender stem tissues, i.e., achieving reliable severing while suppressing non-ideal failure modes such as compression, tearing, and pulling. The cutting of plant stems is fundamentally a mechanical response process jointly governed by material properties, geometric scale, and loading boundary conditions, and is influenced by the coupled effects of tissue structure, moisture status, stem dimensions, support conditions, and blade motion mode, thus exhibiting pronounced material–structure synergy [7,8,9,10,11,12,13,14]. Compared with mature stalks or highly lignified fibrous stems, vegetable sweet potato shoot tips are characterized by high tenderness, high moisture content, and low fiber development; accordingly, their mechanical behavior and cutting-load boundaries are distinctly different, and existing cutting parameters for other crops cannot be directly applied. Previous studies on the mechanical properties of sweet potato vines and stems have provided important references for sweet-potato-related equipment design, but their research objects and operational targets are mainly oriented toward vine handling at the storage-root harvest stage, and therefore still differ from the “suitable harvest” cutting scenario for tender vegetable shoot tips [15].

Research on plant material cutting mechanics has developed a relatively mature methodological foundation, and related studies have systematically clarified the effects of blade geometry, cutting mode, material moisture content, and stem scale on peak load, cutting energy consumption, and mechanical response curves [9,10,11,12,13,14]. At the same time, recent studies on agricultural robotics and experimental evaluation of agricultural machinery indicate that successful mechanization depends not only on the sensing or execution unit itself, but also on whether a consistent framework can be established linking material parameters, operational thresholds, and control strategies, so that field-based empirical rules can be translated into engineering-usable parameter boundaries [16,17,18,19,20]. In the field of sweet potato mechanization, available studies and recent reviews remain largely oriented toward storage-root harvesting, conveying, and separation, whereas repeated harvesting of tender shoot tips is still insufficiently studied from the perspective of engineering parameterization [21,22]. In particular, there is still a lack of systematic studies establishing a direct mapping between field-operable harvest criteria, such as shoot-tip length, and equipment design inputs, such as maximum shear load, design margin, and control thresholds. This gap prevents the formation of a closed loop among harvest standards, material characteristics, and equipment design, thereby constraining the engineering advancement of mechanized harvesting of vegetable sweet potato shoot tips.

In response to the above industrial demand and research gap, this study examined whether a field-operable shoot-tip length standard can serve as an effective engineering criterion for mechanized harvesting of vegetable sweet potato shoot tips. Here, “suitable harvest” is not used to denote a universal agronomic maturity stage, but rather an engineering-feasible harvest length window within which cutting can be completed with an acceptable load margin under a given equipment condition. Under unified experimental conditions, five shoot-tip length levels were established for three vegetable sweet potato cultivars, and maximum shear force, stem diameter, and moisture content were measured systematically. Because the mechanical tests were conducted on short stem segments sampled from a fixed internodal position relative to the apex, the evaluated length factor should be interpreted primarily as an operational harvest criterion and only secondarily as a variable partly associated with tissue position along the stem. Methodologically, within-cultivar one-way ANOVA, pooled two-way ANOVA, and stem-diameter-adjusted ANCOVA were used to distinguish overall length-related patterns, cultivar differences, and geometric-scale effects. In addition, quantile-based design loads were constructed to provide preliminary engineering inputs for cutting-component verification, drive selection, and control-threshold setting, and to enable back-calculation of a feasible harvest length window under a specified allowable load limit. In this way, the study advances harvest decision-making for vegetable sweet potato shoot tips from an experience-based practice toward a parameter-based engineering framework.

## 2. Results

### 2.1. Cultivar Differences in Moisture Content

**Figure 1:**
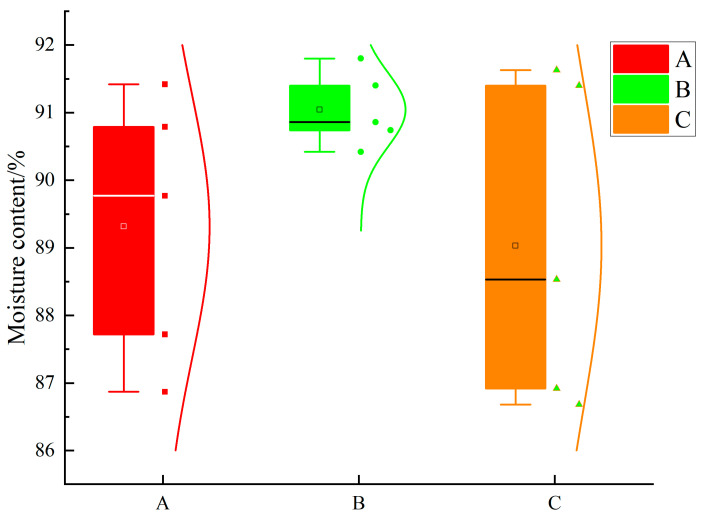
Boxplot of moisture content of the three cultivars. A is ‘Fu 23’; B is ‘Fu 18’; C is ‘HD-V4’. Squares indicate mean values. Because no significant difference was detected among cultivars (*p* > 0.05), identical statistical notation was used across groups.

The moisture content of samples from all three cultivars fell within a high-moisture range (Table 1; Figure 1). A one-way ANOVA indicated that differences in moisture content among cultivars were not significant (*p* = 0.204), suggesting that no systematic moisture bias existed among the samples in this batch. Accordingly, moisture content was used as a background descriptor of material status to support comparability of the subsequent shear-mechanics analysis, rather than as a primary explanatory variable of cutting-force variation.

### 2.2. Descriptive Statistics of Maximum Shear Force, Stem Diameter, and Nominal Shear Stress

Across cultivars and shoot-tip length levels, stem diameter and maximum shear force generally showed a coordinated trend. After normalizing the maximum shear force to the cross-sectional area to obtain the nominal shear stress, however, the pattern of between-cultivar differences changed (Table 2). As shown in Table 2, cultivar B generally had larger stem diameters and a correspondingly higher mean maximum shear force. However, when expressed as nominal shear stress, its values were not dominant, indicating that differences in absolute load were not determined solely by material resistance but were jointly influenced by geometric scale and tissue mechanical behavior. This result provides a basis for subsequent ANCOVA to partition the contribution of stem diameter.

#### 2.2.1. Analysis of Shear-Test Results for ‘Fu 23’

For ‘Fu 23’ (cultivar A), the mean maximum shear force across length levels showed an overall increasing trend, although not strictly monotonic (Table 2). The mean load was relatively low at 10 cm (45.159 ± 5.451 N) and increased overall to approximately 57–64 N within the 15–30 cm range. The nominal-shear-stress data in Table 2 further show relatively high mean values at 15 cm and 25 cm, suggesting that load variation at some length levels in this cultivar cannot be attributed to stem diameter alone and may also be associated with differences in tissue structure. It should be noted that the evaluated length level in this study is partly linked to the anatomical position of the sampled tissue along the stem; therefore, the observed load differences should not be interpreted as a purely geometric length effect alone.

**Figure 2:**
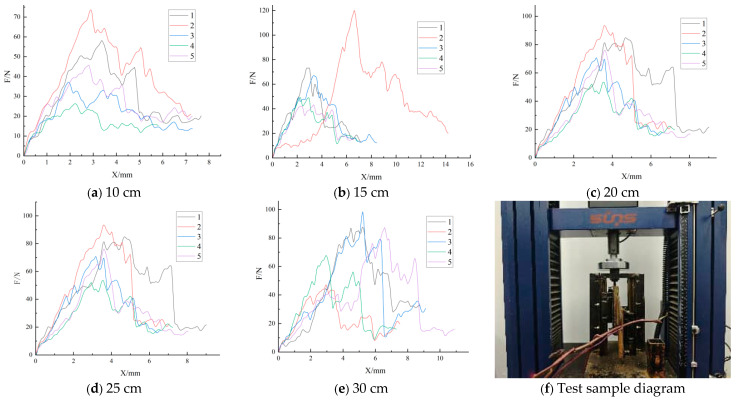
Shear-test results for ‘Fu 23’. Panels (**a**–**e**) show the force–displacement response trends of the five replicate trials at 10, 15, 20, 25, and 30 cm, respectively; panel (**f**) shows the corresponding specimen morphology and sampling/cutting context.

Figure 2 shows that clear force–displacement response peaks were obtained at all length levels, but both peak magnitude and curve shape varied among samples, consistent with the standard-error levels reported in Table 2. Overall, ‘Fu 23’ exhibited an intermediate load range, and the direction of the length-related trend was relatively consistent, although within-group variation still required formal statistical testing through a subsequent ANOVA.

#### 2.2.2. Analysis of Shear-Test Results for ‘Fu 18’

‘Fu 18’ (cultivar B) exhibited comparatively larger stem diameters among the three cultivars and a generally higher mean maximum shear force (Table 2). Its mean maximum shear force increased from 48.142 ± 8.218 N at 10 cm to 77.684 ± 9.111 N at 30 cm. The relatively large standard errors at 15 cm and 20 cm suggest substantial between-sample variability at some length levels for this cultivar.

**Figure 3:**
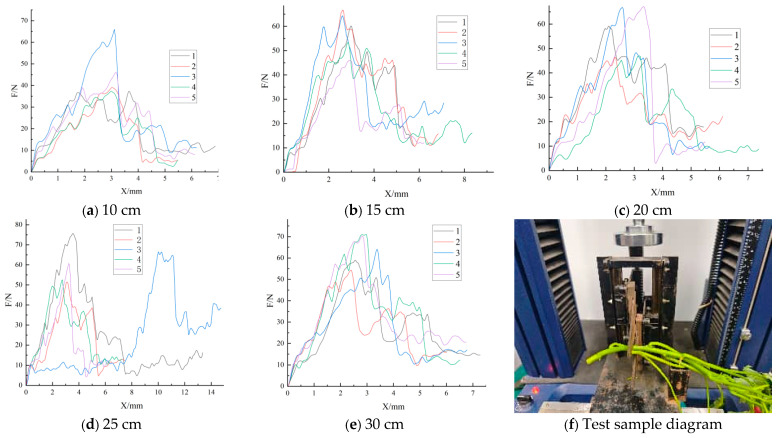
Shear-test results for ‘Fu 18’. Panels (**a**–**e**) show the force–displacement response trends of the five replicate trials at 10, 15, 20, 25, and 30 cm, respectively; panel (**f**) shows the corresponding specimen morphology and sampling/cutting context.

The nominal-shear-stress data in Table 2 further show that, although ‘Fu 18’ had relatively high absolute loads, its mean nominal shear stress was generally lower, supporting the inference that the elevated loads were strongly amplified by larger cross-sectional dimensions. Figure 3 shows that the peak-load distribution was relatively broad at some length levels, especially at 15 cm and 20 cm, which is consistent with the comparatively large standard errors reported in Table 2. It should be noted that the evaluated length level in this study is partly linked to the anatomical position of the sampled tissue along the stem; therefore, the observed load differences should not be interpreted as a purely geometric length effect alone.

#### 2.2.3. Analysis of Shear-Test Results for ‘HD-V4’

For ‘HD-V4’ (cultivar C), the mean maximum shear force across length levels showed a non-monotonic pattern of increasing first and then decreasing (Table 2): it increased from 57.202 ± 3.213 N at 10 cm to 77.170 ± 10.413 N at 25 cm, and then dropped to 56.366 ± 2.977 N at 30 cm. This suggests that, for cultivar C, the relationship between a greater evaluated length level and a greater load does not hold across the full range, and that the observed response may be jointly influenced by tissue position, local structure at the shear location, and stem diameter.

**Figure 4:**
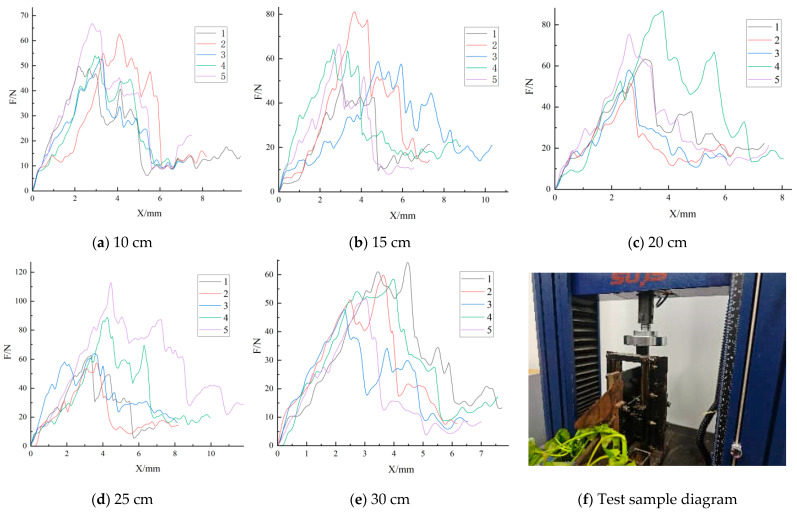
Shear-test results for ‘HD-V4’. Panels (**a**–**e**) show the force–displacement response trends of the five replicate trials at 10, 15, 20, 25, and 30 cm, respectively; panel (**f**) shows the corresponding specimen morphology and sampling/cutting context.

Figure 4 shows that some samples at 25 cm exhibited relatively high peak loads, whereas the overall peak level declined at 30 cm, consistent with the descriptive statistics in Table 2. This non-monotonic pattern also provides a phenomenon-level explanation for the subsequent within-cultivar one-way ANOVA result in which the length effect did not reach significance. It should be noted that the evaluated length level in this study is partly linked to the anatomical position of the sampled tissue along the stem; therefore, the observed load differences should not be interpreted as a purely geometric length effect alone.

### 2.3. ANOVA of Length Effects Within Cultivars (Within-Group Analysis)

To examine the stability of the length effect within each cultivar, a one-way ANOVA was performed separately for cultivars A, B, and C (Table 3). As shown in Table 3, the length effect did not reach statistical significance at the 0.05 level in any of the three cultivars. Cultivar A had the *p* value closest to the significance threshold and the highest effect size (*η*^2^), indicating a relatively stronger trend in its length effect.

Taken together with the patterns in Table 2 and Figure 2, Figure 3 and Figure 4, the non-significant within-cultivar results should not be interpreted as definitive evidence that length has no influence on shear load. Rather, they indicate that, under the present sample size and within-group variability, the detectability of length-related differences was limited and cultivar-dependent. Therefore, the within-cultivar analyses are interpreted here as supportive pattern-level evidence, whereas stronger inference is reserved for the pooled analysis and the covariate-adjusted decomposition.

### 2.4. Overall Two-Way ANOVA (Overall Effects)

After pooling the data from all three cultivars, a two-way ANOVA was performed using maximum shear force as the response variable (Table 4). As shown in Table 4, both the cultivar main effect and the length main effect were significant, whereas the cultivar × length interaction effect was not significant. This indicates that, at the overall sample level, the differences in maximum shear force were jointly influenced by cultivar and length, but the response patterns to length did not differ significantly among cultivars.

### 2.5. Geometric-Scale Decomposition: Contribution of Stem Diameter to Load Differences (ANCOVA and Regression)

To identify the contribution of geometric scale to the differences in maximum shear force, stem diameter was introduced as a covariate in the pooled analysis, and an ANCOVA was performed within a common-slope framework (Table 5). As shown in Table 5, the stem-diameter effect was significant, the length effect remained significant, and the cultivar main effect became non-significant after controlling for stem diameter. These results indicate that a substantial portion of the between-cultivar differences in maximum shear force can be explained by stem diameter, i.e., geometric scale. Once the effect of geometric scale is accounted for, the independent explanatory power of cultivar identity is substantially weakened. Consistent with the descriptive statistics in Table 2, the relatively high absolute loads observed in ‘Fu 18’ therefore appear to be strongly associated with geometric-scale amplification. Because the adjusted interpretation depends on the common-slope assumption, the ANCOVA results should be interpreted within the scope of that model framework and with due caution regarding the present sample size.

Consistent with the descriptive statistics in Table 2, the relatively high absolute loads observed in ‘Fu 18’ showed a strong geometric-scale amplification effect. However, this adjusted interpretation should be understood within the ANCOVA framework and with appropriate caution regarding model assumptions and the present sample size.

### 2.6. Engineering Output of Quantile-Based Design Loads and Suitable Harvest Length Windows

Based on the quantile statistics for each cultivar at each length level, the median load (*Q*_0.5_), the upper-quantile load (*Q*_0.95_), and the design load (*F*_design_) were calculated (Table 6). As shown in Table 6, design loads differed substantially across the cultivar × length combinations, indicating that designing the cutting mechanism based only on the mean load may underestimate peak risk under the upper-tail operating conditions. Because the cultivar × length group sample sizes were limited, the estimated *Q*_0.95_ values should be interpreted as conservative engineering indicators derived from the available sample, rather than as highly precise population upper-tail parameters.

Specifically, cultivar A showed a relatively stable design-load range across the length levels; cultivar B had the highest design load at 15 cm, with 30 cm and 20 cm also remaining relatively high; and cultivar C showed a marked increase in the design load at 25 cm, followed by a decline at 30 cm. These results are consistent with the descriptive statistics in Table 2 and the curve patterns in Figure 2, Figure 3 and Figure 4, indicating that the suitable harvest length is not a simple monotonic relationship, but instead reflects the combined effects of cultivar differences and upper-tail risk characteristics.

Accordingly, in engineering applications, Table 6 can be used to establish a decision rule for suitable harvest length windows: under a given allowable equipment load limit (*F*_allow_), the set of length levels satisfying the design-load constraint can be defined as the suitable harvest length window under that equipment condition. The upper-quantile loads and the design loads in Table 6 therefore provide preliminary engineering input parameters for this determination.

## 3. Discussion

### 3.1. Engineering Meaning of the “Suitable Harvest” Concept and the Scope in This Study

In this study, “suitable harvest” refers to the shoot-tip length range within which cutting can be completed with an acceptable load margin under a given equipment capacity and operating constraint. This definition is used as an engineering feasibility criterion rather than a comprehensive agronomic optimum. It emphasizes cutting stability, overload risk, and engineering implementability, rather than optimal product quality, yield, or total economic return. Under this definition, the moisture content serves mainly to confirm background comparability among groups, the maximum shear force and the upper-quantile design load provide the main engineering inputs for component verification and threshold setting, and the shoot-tip length functions as the most field-operable and standardizable management variable.

At the same time, length is not treated here as a purely geometric determinant of cutting behavior. Because the shear tests were conducted on short stem segments sampled from a fixed internodal position relative to the apex, the evaluated harvest length factor is partly linked to the anatomical position along the stem. Accordingly, the observed load differences may reflect not only the harvest length as a management variable, but also positional differences in the stem structure, including local diameter, tissue composition, and structural development. Under the present laboratory conditions, these factors together support an operational interpretation of “suitable harvest” as a testable engineering criterion.

### 3.2. Statistical Interpretation of Cultivar Differences, Length Standards, and Geometric-Scale Effects

The pooled analysis detected a significant cultivar main effect, whereas the cultivar effect became non-significant after stem diameter was introduced as a covariate. This pattern indicates that a substantial part of the absolute load difference among cultivars is associated with geometric-scale variation, especially stem diameter. This interpretation is consistent with previous studies showing that cutting and shearing loads scale strongly with stem size and cross-sectional dimensions [13,14]. From the perspective of mechanical design and control, cultivar identity alone is therefore less informative than a parameterization based on stem-diameter distribution together with an operational harvest length standard.

The apparent difference between the within-cultivar analyses and the pooled model is also statistically reasonable. Under biological-material testing conditions, relatively high individual variability and limited within-group sample size can reduce the detectability of factor effects. The non-significant within-cultivar results should therefore not be interpreted as definitive evidence that length has no influence on shear load. Rather, they indicate limited statistical detectability under the present sample size and variability structure. Accordingly, the present results are interpreted mainly at the overall-pattern level, whereas cultivar-specific inference is kept appropriately conservative.

### 3.3. Implications for Mechanized Harvesting Equipment Development

The quantile-based design loads for maximum shear force provide one of the most practically useful engineering outputs of this study because upper-tail loads combined with a safety factor are more consistent with reliability-oriented design than the mean values or single observed peaks. These design loads can provide preliminary guidance for actuator thrust or torque estimation, drive-unit selection, power-margin allocation, and load-limit setting for blade and support-component verification. At the control level, translating design loads into cutting thresholds may help reduce incomplete cutting, repeated cutting, and overload shutdown, thereby improving operational stability.

The nominal-shear-stress results also indicate that a single absolute load threshold may not be sufficiently adaptable across cultivars or operating scenarios. Incorporating the stem diameter as an online measurable or estimable variable is more consistent with sensing-assisted parameter adaptation in agricultural robotics [16,17,18]. From an engineering perspective, such load-derived thresholds are relevant not only to actuator sizing but also to the durability and wear-related reliability of machine elements under repeated service conditions [23]. However, these implications should be interpreted cautiously. The present experiments were conducted under controlled quasi-static laboratory conditions and did not explicitly include several field-relevant factors, such as cutting speed, blade wear, support variability, vibration, and repeated plant–machine interactions. Therefore, the present study provides a laboratory-based basis for preliminary static verification and future control-oriented extension, rather than a direct one-to-one prediction of field operating loads.

### 3.4. Role in a Sustainable Multi-Harvest Production System

Vegetable sweet potato shoot tips are harvested repeatedly, and frequent harvest windows make production efficiency highly dependent on stable and executable harvest standards. If the harvest length remains primarily experience-based, specification variability, unstable operational quality, and higher labor-organization costs may weaken supply stability in multi-harvest systems. The present results provide a practical basis for converting a field-operable harvest rule into quantified engineering parameters that can be linked to equipment capacity and control thresholds. More broadly, the translation of laboratory-derived parameter ranges into field-operable machinery settings still requires validation under realistic crop–machine operating systems [24]. Therefore, matching design loads to equipment capacity offers a repeatable way to define machine-specific suitable harvest windows, although such windows should still be regarded as conditional engineering ranges rather than universal field prescriptions.

### 3.5. Limitations and Future Research

This study has several limitations. First, the experiments were performed under quasi-static shearing and did not systematically include key operating factors such as cutting speed, blade wear, support mode, and clamping stiffness. Second, because the shear-test specimens were taken from a fixed internodal position relative to the apex, the evaluated harvest length factor is partly confounded with anatomical position along the stem. Third, the upper-tail design loads were derived from relatively small cultivar × length group samples, and the estimated 95th percentiles should therefore be interpreted as preliminary conservative engineering indicators rather than highly precise population extremes. Fourth, the material scope was restricted to the three cultivars under a single-batch test condition.

Future work should expand cultivar and eco-regional sampling, incorporate dynamic cutting conditions and key mechanical factors, test broader sampling positions along the stem, and validate a closed-loop prototype workflow of “stem diameter estimation–load prediction–adaptive parameter control,” thereby strengthening the transferability of the proposed engineering criteria.

## 4. Materials and Methods

### 4.1. Experimental Materials and Cultivation Management

Three vegetable sweet potato cultivars were used as test materials and are denoted as A, B, and C. Cultivar A was ‘Fu 23’, characterized by red shoot tips, relatively slender and soft stems, and fewer lateral buds. Cultivar B was ‘Fu 18’, characterized by green shoot tips, notched leaves, and soft stems. Cultivar C was ‘HD-V4’, characterized by green shoot tips, standard cordate (heart-shaped) leaves, and relatively more fibrous and stiffer stems.

**Figure 5:**
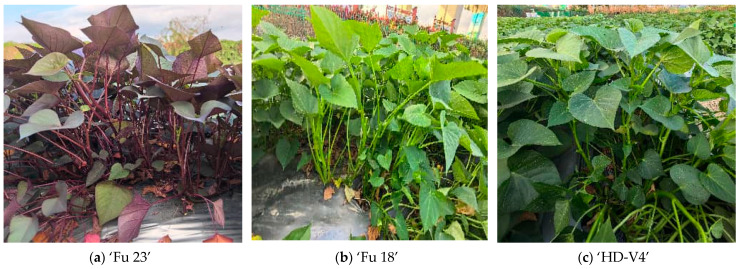
Growth morphology of the three cultivars at sampling: (**a**) Cultivar A (‘Fu 23’), (**b**) Cultivar B (‘Fu 18’), and (**c**) Cultivar C (‘HD-V4’).

The experiment was conducted at the Hainan University experimental base, Hainan, China, and sampling was carried out on 2 February 2026. At sampling, the plants had reached the stage at which shoot tips are routinely harvested by hand in local production practice; therefore, the sampled materials represented the manually harvestable stage considered in this study. Mother plants were selected based on uniform growth status, vigorous vegetative growth, and the absence of obvious pest or disease symptoms. Only visually marketable shoot tips showing uniform external maturity and no visible mechanical or pest damage were included. To reduce within-plant correlation and facilitate traceability, samples of each cultivar were collected from at least five mother plants. Field management, including water and fertilizer application, was kept consistent during the experimental period. Sampling was performed under the same cultivation batch and management background to minimize non-experimental variation among cultivars. The growth morphology of the three cultivars at sampling is shown in Figure 5.

### 4.2. Experimental Instruments and Consumables

Mechanical testing was conducted using a DWD-type electronic universal testing machine (Sansi Co., Ltd., Shenzhen, China) equipped with a 5 kN load cell. The machine had an accuracy class of 0.5, a displacement resolution of 0.01 mm, and an adjustable loading rate of 0.01–500 mm min^−1^. A single-blade shear fixture was mounted on the testing machine, and the blade edge was positioned reproducibly for each test. Force calibration and verification of the testing machine followed the relevant practice for testing machines [25].

**Figure 6:**
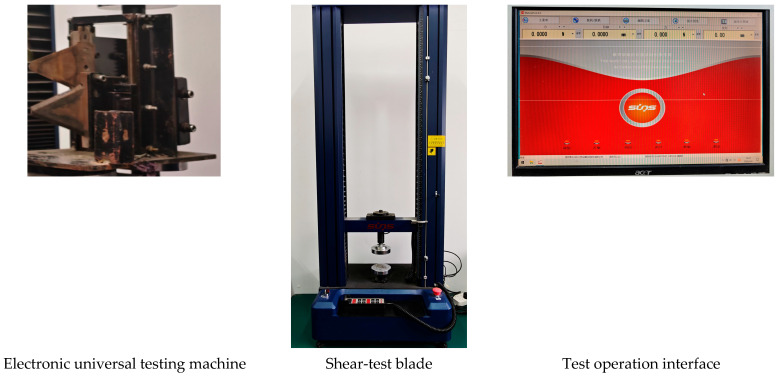
Experimental apparatus configuration and shear-testing setup.

**Figure 7:**
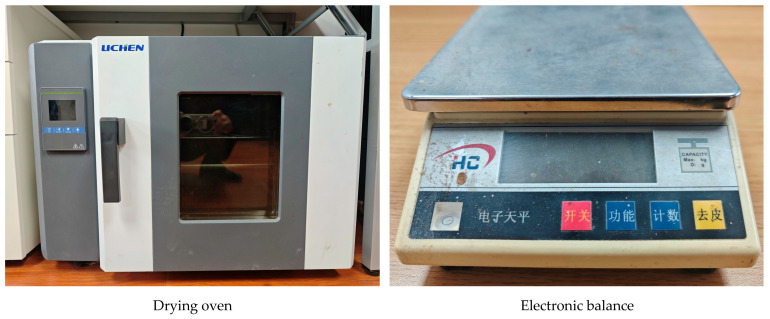
Workflow of moisture content measurement, including sample weighing and drying-related procedures.

Moisture content determination was performed using a DGF30/7-IA electric blast drying oven (LICHEN, Zhejiang Lichen Instrument Technology Co., Ltd., Shaoxing, China; temperature range, 0–300 °C; 220 V). Fresh and oven-dry masses were measured using a top-loading electronic balance (HC, Shanghai Huachao Industrial Co., Ltd., Shanghai, China; range 100 g, accuracy 0.0001 g). Stem diameter was measured using a Pro’sKit high-precision digital vernier caliper (Prokit’s Industries Co., Ltd., New Taipei City, Taiwan; range, 150 mm; accuracy, 0.01 mm). Sampling and storage were carried out using sealed moisture-retaining containers and a labeling system to ensure that samples were tested as soon as possible after sampling and that sample IDs corresponded one-to-one with curve files. Figure 6 and Figure 7 are provided to illustrate the apparatus configuration and the workflow of specimen processing, weighing, drying, and mechanical testing, thereby improving the transparency and reproducibility of the experimental procedure.

### 4.3. Experimental Design and Sample Size

The shear test followed a two-factor design. Factor 1 was cultivar (A, B, C), and Factor 2 was shoot-tip length level (10, 15, 20, 25, and 30 cm). Each cultivar × length combination included five replicates. The experimental unit was one individual shoot-tip sample, giving a total sample size of 75. The testing order was randomized to reduce systematic effects caused by time drift and operator factors. For the moisture content test, samples were grouped by cultivar, with five replicates per cultivar and a total sample size of 15. The experimental design factors and levels are shown in Table 7.

### 4.4. Sampling, Pretreatment, and Data Traceability

Using the shoot-tip apex as the reference point, samples were cut downward along the stem axis to total lengths of 10, 15, 20, 25, and 30 cm, with the length error controlled within ±0.5 cm. Accordingly, sample length in this study was defined from the shoot-tip apex rather than from the soil surface or the basal trunk. Mechanical test specimens were consistently taken from a fixed internodal region near the basal cut end of each sampled shoot tip to ensure comparability among different length levels. The harvest length factor used in this study therefore represents an operational length category that is partly associated with the anatomical position of the sampled tissue along the stem. This definition was adopted to align the evaluated length levels with a field-operable harvest rule for shoot-tip collection.

**Figure 8:**
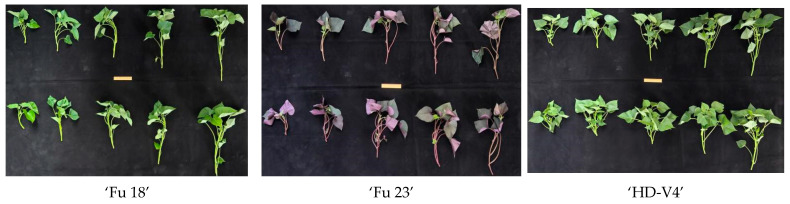
Sampling scheme at different shoot-tip length levels.

**Figure 9:**
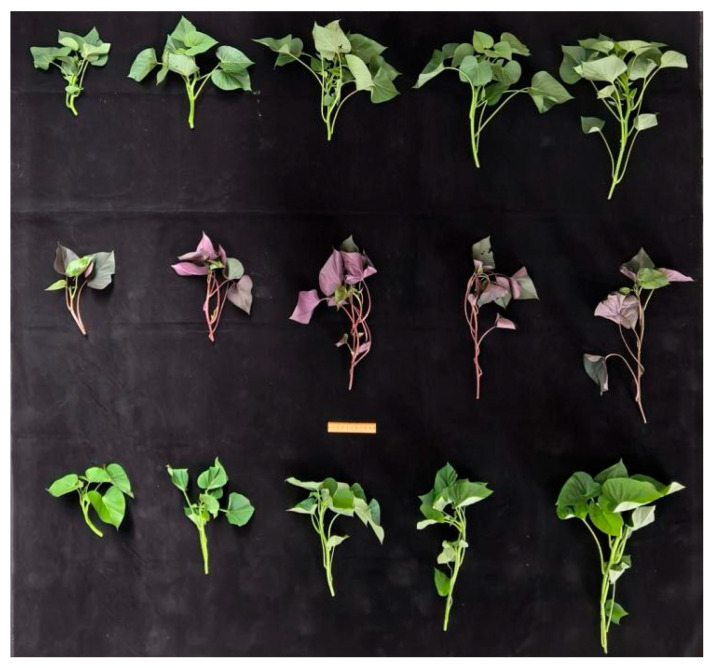
Comparison of representative samples from the three cultivars after collection.

For shear testing, stem segments of approximately 2 cm in length were prepared, and stem diameter was measured at the same position as the shear specimen. After sampling, specimens were placed in moisture-retaining containers, and mechanical testing was completed within 1 h after collection. Figure 8 and Figure 9 illustrate the sampling scheme at different length levels and the post-collection comparison of representative samples among cultivars.

### 4.5. Test Procedure

**Figure 10:**
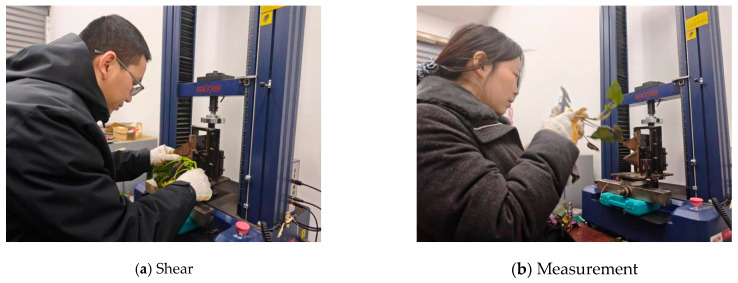
Field sampling context and selection of representative shoot-tip material prior to laboratory testing.

To minimize systematic bias introduced by instrument condition and operator effects, zero calibration, fixture alignment, and blade-condition checks were performed before each testing batch. The crosshead speed was fixed at 10 mm min^−1^ for all shear tests. The shear blade was inspected after each testing batch and was resharpened or replaced whenever visible dulling or edge damage was observed, in order to maintain consistent cutting conditions throughout the experiment. Laboratory temperature during testing was recorded and remained within 22–25 °C. The experimental procedure followed a unified in-house protocol based on constant fixture geometry, constant loading rate, and consistent specimen positioning. Figure 10 documents the field sampling context and the selection of representative shoot-tip material prior to laboratory testing.

### 4.6. Measured Variables and Measurement Methods

#### 4.6.1. Moisture Content

Moisture content was determined by the oven-drying method on a wet basis following the general analytical principle of oven drying [26]. Fresh sample mass was denoted as *M_f_*, and dry sample mass was denoted as *M_d_*. In this study, moisture content was calculated as:

(1) $$M=\frac{M_{f}-M_{d}}{M_{f}}\;\times \;100\%$$

where *M* is moisture content (%), and *M_f_* is fresh mass (g), and *M*_d_ is dry mass (g). Moisture content measurement was performed using the electronic balance and drying oven described in Section 4.2. Five independent replicate samples were evaluated for each cultivar. Drying was continued to constant mass, defined as a difference of less than 0.01 g between two consecutive weighings.

#### 4.6.2. Maximum Shear Force

A single-blade fixture mounted on the electronic universal testing machine was used. A stem segment with uniform length was prepared from the fixed internodal region defined in Section 4.4, placed horizontally and fixed, and cut through at the midpoint by aligning the blade edge with the center of the specimen. The force–displacement curve was recorded and the maximum shear force was extracted.

The maximum shear force was defined as:

(2) $$F_{s,max}\;=\underset{x\in [0,\mathrm{xc}]}{\max}F_{s}(x)$$

where *F_s_*(*x*) is the force–displacement function during shearing, N; *x* is displacement, mm; x_c_ is the displacement at the completion of cutting, mm; and *F_s_*_,max_ is the maximum shear force, N.

#### 4.6.3. Stem Diameter and Nominal Shear Stress

Stem diameter was denoted as d (mm) and measured at the same location as the shear specimen. Two measurements were taken along mutually perpendicular directions and averaged. To partially separate the contribution of geometric scale from the absolute load magnitude, the cross-sectional area *A* and the nominal shear stress τ were calculated as follows:

(3) $$A\;=\;\frac{\pi d^{2}}{4}$$

(4) $$\tau \;=\frac{F_{s,max}}{A}$$

where *A* is in mm^2^, and *τ* is in N·mm^−2^, numerically equivalent to MPa. Because the present test used a single-blade cutting arrangement, the internal stress field in the stem was not spatially uniform. Therefore, *τ* should be interpreted as the nominal shear stress for comparative engineering analysis rather than as a true intrinsic material property.

### 4.7. Statistical Analysis and Engineering Expression of the Suitable Harvest Length Window

#### 4.7.1. Test of Cultivar Differences in Moisture Content

Using the moisture content *M* as the response variable, a one-way ANOVA was applied to test cultivar differences at a significance level of 0.05. This analysis was used to verify the comparability of the moisture background among cultivars.

#### 4.7.2. Overall Analysis: Two-Way ANOVA

Using the maximum shear force as the response variable, the cultivar effect, the length effect, and their interaction were tested:

(5) $$F_{s,max,ijk}\;=\;\mu \;+V_{i}\;+\;L_{j}\;+\;{\left(VL\right)}_{ij}\;+{\;\varepsilon }_{ijk}$$

where *μ* is the overall mean, *V_i_* is the cultivar main effect, *L_j_* is the length main effect, (*VL*)*_ij_* is the interaction effect, and *ε_ijk_* is the random error. Because the interaction effect was not significant, the main effects were interpreted at the overall level.

#### 4.7.3. Within-Group Analysis: Separate Tests of Length Effect for Each Cultivar

A one-way ANOVA of the length level was performed separately within cultivars A, B, and C to evaluate the stability and variability of the length effect within each cultivar. The effect size *η*^2^ was also calculated as an indicator of the within-group explanatory power.

#### 4.7.4. Geometric-Scale Decomposition: ANCOVA and Regression

To identify the contribution of the stem diameter to differences in the shear load, the stem diameter was introduced as a covariate in the pooled analysis and interpreted within a common-slope ANCOVA framework. Because the adjusted interpretation depends on the common-slope assumption between the covariate and the response across factor levels, the ANCOVA results were interpreted only within the scope supported by that framework. In addition, a linear regression model between *F_s,max_* and *d* was fitted to describe the scale dependence of the peak shear load. Statistical analyses were conducted in *R* [27].

#### 4.7.5. Quantile-Based Design Load and the “Suitable Harvest Length Window”

For the distribution of the maximum shear force in each cultivar × length combination, the median *Q*_0.5_ and the 95th percentile *Q*_0.95_ were calculated. A design load was then constructed using the 95th percentile together with a safety factor:

(6) $$F_{\mathrm{design}}\;=\;\gamma \;\times {\;Q}_{0.95}$$

In this study, *γ* = 1.3. To characterize the degree of amplification of the upper-tail loads relative to the median level, a tail amplification coefficient was defined as:

(7) $$K_{\mathrm{tail}}\;=\;\frac{Q_{0.95}}{Q_{0.5}}$$

Because treatment-group sample sizes were limited, *Q*_0.95_ and the resulting design load should be interpreted as conservative engineering indicators derived from the available sample rather than as exact population upper-tail parameters.

Given an allowable upper limit of the equipment design load *F*_allow_, the feasible cultivar–length combinations were defined as those satisfying the design-load constraint. This feasible set can be expressed as:

(8) $$\Omega \left(F_{\mathrm{allow}}\right)\;=\;\{(c,l)|F_{\mathrm{design}}(c,l)\leq F_{\mathrm{allow}}\}$$

where Ω(*F*_allow_) denotes the engineering expression of the “suitable harvest length window” under a specified equipment load constraint.

It should be noted that moisture content data were not stratified by length level. Therefore, moisture content was used in the window determination only to describe the background consistency of material moisture status, rather than to explain moisture effects along the length gradient.

## 5. Conclusions

Using a cultivar × length factorial design, this study characterized cutting-load variability in vegetable sweet potato shoot tips and identified the variables that most directly govern the peak shear demand. The comparable moisture background across cultivars (*p* > 0.05) supports the interpretation that the observed load differences were not driven by systematic moisture bias. Across the pooled dataset, the maximum shear force varied with length, and the absence of a significant cultivar × length interaction suggests that length-based field rules can be formulated at the overall-pattern level without cultivar-specific response patterns dominating the trend. Importantly, once the stem diameter was accounted for, cultivar identity no longer explained additional variance in the peak load, highlighting geometric scale, rather than the cultivar label, as the more transferable basis for parameter setting.

From an engineering perspective, converting shear-force distributions into upper-tail design loads (95th percentile with *γ* = 1.3) provides a practical basis for preliminary sizing of cutting components, drive-margin allocation, and overload-threshold setting, and enables an explicit “suitable harvest length window” to be determined under a given allowable load limit. Here, “suitable harvest” is interpreted as an engineering-feasible harvest length window rather than a universal agronomic optimum. Collectively, the present work provides a practical bridge between field-operable harvest standards and equipment-oriented load boundaries under controlled laboratory conditions. Future research should expand cultivar and seasonal coverage, test broader sampling positions along the stem, and incorporate dynamic cutting conditions such as speed, blade sharpness decay, and support or fixture effects in order to validate and refine these criteria under field-relevant operating regimes.

## Figures and Tables

**Table 1:** Descriptive statistics of moisture content and test of cultivar differences.

Cultivar	*n* (Replicates)	Mean Moisture Content (%)	Standard Error (%)
A	5	89.31	0.87
B	5	91.04	0.25
C	5	89.03	1.06

**Table 2:** Stem diameter, maximum shear force, and nominal shear stress at different cultivar × length combinations (mean ± standard error).

Cultivar	Length (cm)	Stem Diameter (mm)	Maximum Shear Force (N)	Nominal Shear Stress (MPa)
A	10	5.498 ± 0.281	45.159 ± 5.451	2.050 ± 0.501
A	15	4.592 ± 0.177	58.117 ± 3.785	3.522 ± 0.205
A	20	5.300 ± 0.277	57.300 ± 4.570	2.770 ± 0.538
A	25	4.854 ± 0.404	61.381 ± 4.509	3.534 ± 0.493
A	30	5.048 ± 0.124	63.729 ± 3.208	3.212 ± 0.240
B	10	6.260 ± 0.332	48.142 ± 8.218	1.530 ± 0.180
B	15	7.050 ± 0.422	70.600 ± 13.552	1.810 ± 0.301
B	20	6.748 ± 0.364	75.635 ± 6.785	2.119 ± 0.131
B	25	7.138 ± 0.627	74.195 ± 3.237	1.954 ± 0.208
B	30	7.188 ± 0.486	77.684 ± 9.111	1.935 ± 0.230
C	10	5.486 ± 0.207	57.202 ± 3.213	2.488 ± 0.293
C	15	6.032 ± 0.255	63.895 ± 5.290	2.252 ± 0.186
C	20	5.656 ± 0.514	67.034 ± 6.291	2.765 ± 0.277
C	25	5.914 ± 0.500	77.170 ± 10.413	2.841 ± 0.264
C	30	5.196 ± 0.227	56.366 ± 2.977	2.690 ± 0.191

Note: Cultivar A = ‘Fu 23’; Cultivar B = ‘Fu 18’; Cultivar C = ‘HD-V4’.

**Table 3:** One-way ANOVA performed separately for cultivars A, B, and C.

Cultivar	df (Length)	df (Error)	*F*	*p*	*η* ^2^
A	4	20	2.694	0.060	0.35
B	4	20	1.868	0.156	0.272
C	4	20	1.836	0.162	0.269

**Table 4:** Two-way ANOVA with maximum shear force as the response variable.

Source of Variation	f	*F*	*p*
Cultivar	2	4.085	0.0217
Length	4	4.112	0.0052
Cultivar × length interaction	8	0.905	0.5181

**Table 5:** ANCOVA with stem diameter as a covariate.

Source of Variation	f	*F*	*p*
Cultivar	2	0.750	0.4764
Length	4	4.474	0.0029
Stem diameter	1	19.431	0.000039

**Table 6:** Quantile statistics for each cultivar at each length level.

Cultivar	Length (cm)	*Q*_0.5_ (N)	*Q*_0.95_ (N)	*F*_design_ (N)
A	10	39.0908	62.0064	80.6083
A	15	60.1450	66.2432	86.1161
A	20	59.1811	67.0927	87.2206
A	25	60.7507	73.8181	95.9636
A	30	64.1284	70.8563	92.1131
B	10	45.6636	70.4687	91.6093
B	15	66.9475	110.7677	143.9980
B	20	75.9999	91.7312	119.2505
B	25	74.2635	82.4198	107.1458
B	30	87.3662	96.3368	125.2378
C	10	54.0246	66.0176	85.8229
C	15	64.2096	78.2317	101.7012
C	20	63.3629	84.5056	109.8573
C	25	63.9416	107.9884	140.3849
C	30	58.4454	63.3779	82.3913

**Table 7:** Experimental design factors and levels.

Factor	Symbol	No. of Levels	Level Definition
Cultivar		3	A; B; C
Length level	L	5	10, 15, 20, 25, 30 cm

## Data Availability

The data presented in this study are available on-demand from the correspondence author at (shenhaiyang@caas.cn).
